# The critical role of mobile phase pH in the performance of oligonucleotide ion-pair liquid chromatography–mass spectrometry methods

**DOI:** 10.2144/fsoa-2021-0084

**Published:** 2021-10-22

**Authors:** Guilherme J Guimaraes, Michael G Bartlett

**Affiliations:** 1 Department of Pharmaceutical & Biomedical Sciences, College of Pharmacy, University of Georgia, Athens, GA, USA

Liquid chromatography in conjunction with mass spectrometry (LC–MS) has become a major technique in the quality control and bioanalysis of oligonucleotides [1–8]. Over the past 30 years, there have been significant efforts made to improve and optimize mobile phase conditions to increase method performance. The approach that has been the most widely used is ion-pair chromatography using an alkylamine with a fluoroalcohol. This was initially triethylamine and hexafluoroisopropanol but there are many other combinations of alkylamines and fluoroalcohols that have shown improved performance, for specific applications [9–11]. There have also been studies that have evaluated the concentrations of various alkylamines and fluoroalcohols, both of which will cause ion suppression during electrospray ionization at elevated concentrations [12–16].

Another chromatographic parameter that has received considerable attention is the column temperature. It has been shown that oligonucleotides can adopt complex folded structures or even duplexes during analysis. These higher order structures will alter chromatographic retention times and electrospray ionization charge state distributions [17–19]. In order to remove these often less predictable changes, most methods operate at temperatures of 50–70°C. This is well above typical annealing temperatures for oligonucleotides and ensures that all of the structures are linear [20]. Being in a linear form will cause oligonucleotides to elute based on their size in the alkylamine/fluoroalcohol system, which facilitates impurity and metabolite determination.

The main organic solvent used in oligonucleotide LC–MS analysis has been methanol. This solvent was initially used due to the increased solubility of fluoroalcohols in it relative to acetonitrile. However, at lower concentrations of fluoroalcohols, acetonitrile can be used. It does appear that generally, methanol provides improved electrospray ionization efficiency relative to acetonitrile. However, this has to be balanced against the solubility of some of the more hydrophobic alkylamines in methanol. Therefore, if an application needed to use alkylamines like hexylamine or octylamine, acetonitrile would likely be a better choice. Chen and co-workers suggest that organic solvent low dielectric constant explains ethanol and methanol higher signal intensity in comparison with acetonitrile when fluoroalcohol hexafluoroisopropanol (HFIP) is implemented [21]. When first implemented, the alkylamine/fluoalcohol ion-pair system consisted of 400 mM fluoroalcohol and 100 mM alkylamine [10,22]. Due to ion suppression, much lower concentrations have been used with the fluoroalcohol concentration ranging between 25 and 50 mM and alkylamine concentration ranging between 10 and 15 mM.

Despite the significant efforts that have been made to systematically evaluate and optimize these mobile phase components, one of the most influential parameters in liquid chromatography has never been seriously addressed. This is particularly noteworthy since ion-pair chromatography relies on the ability to effectively control pH in order to maintain efficient ionic interactions during separations. There is growing evidence that many of the most significant challenges in LC–MS of oligonucleotides are greatly influenced by the pH of the mobile phase. Among these challenges the two that appear to be the most influenced by pH are mobile phase stability (also known as aging) and nonspecific adsorption.

In order to discuss these issues in depth, a thorough understanding of several key issues is required. These include: the properties of the various mobile phase components; the influence of materials used in LC–MS; how the chemicals and materials used are impacted by pH; and the separation mechanisms that the analytes are undergoing within the chromatographic column.

## The alkylamine/fluoroalcohol mobile phase system

The alkylamine/fluoroalcohol mobile phase system was first proposed by Hancock and co-workers and was a major advance over the alkylamine acetate mobile phases used previously [10]. Acetate has a high Henry’s Law partition coefficient relative to the other components of the mobile phase, which leads to significant ion suppression during electrospray ionization [21]. McGinnis and co-workers identified relative low Henry constant values in individual alkylamines to correlate with higher signal intensities [12]. Additionally, Chen and co-workers observed the same correlation applies to acidic modifiers such as formic and acetic acid. The idea of using the hexafluoroisopropanol as a replacement was to add a weak acid, that is significantly more volatile than acetate, to help maintain the ion-pair buffering system but reduce ion suppression [10,22]. This simple substitution leads to more than an order of magnitude improvement in the electrospray response for oligonucleotides, which explains its wide adoption.

Over the past decade, there have been many incremental improvements on this mobile phase involving the use of different alkylamines and fluoroalcohols [12,21,23,24]. This has led to an increased understanding of the role that each of these play in both the chromatographic separation and the electrospray desorption of oligonucleotides. Interestingly, there does not appear to be any universal best combination of alkylamine and fluoroalcohol for LC–MS experiments. However, Basiri and co-workers have developed a model that helps to predict the best alkylamine for a particular oligonucleotide sequence and further research by Basiri and co-workers has shown that the optimal fluoroalcohol tracks with the hydrophobicity of the alkylamine [9,11]. Additionally, Gong and co-workers recommend ion-pair composition based on oligonucleotide size [25].

During chromatography, the alkylamine intercalates into the C_18_ stationary phase modifying its properties and introducing an ionic interaction between the oligonucleotide and the immobilized alkylamine. The fluoroalcohol acts as a counter ion to the alkylamine, since it is charged at the pH of most mobile phases. Work by Kipper and co-workers has shown that fluoroalcohols can also alter the stationary phase [26]. For many acidic compounds, this leads to more favorable stationary phase/mobile phase distribution constants providing modest improvements in overall chromatography for these analytes.

During electrospray desorption, the mobile phase components work together to bring oligonucleotides to the surface of the droplets where they can be desorbed into the gas-phase. Oligonucleotides are among the most hydrophilic of biomolecules. This causes them to be more stable when completely surrounded by water and; therefore, they are concentrated in the center of droplets. The positively-charged alkylamines are able to associate with the negatively-charged backbone and help to transiently neutralize these molecules making their transport to the surface of the droplet more favorable. Oligonucleotide migration to the droplet surface is also facilitated by increased droplet hydrophobicity caused by the presence of alkylamines and the elevated pH favors negative ionization while controlling cation adduction [12,14]. Muddiman and co-workers have shown that these alkylamine–oligonucleotide complexes are stable into the gas-phase where they exist as proton bound dimers [27]. The phosphate backbone and alkylamine ion-pairs have similar gas-phase proton affinities and; therefore, these systems tend to lead to roughly 50% charging of oligonucleotides. The fluoroalcohol provides some assistance in the surface desorption of oligonucleotides from electrospray droplets by helping to reduce droplet surface tension.

Overall, the alkylamnes and fluoroalcohols have positive effects on both the chromatography and electrospray desorption. However, this places a burden on the user to make sure to balance the positive effects of these reagents against their deleterious effects. Both alkylamines and fluoroalcohols can cause ion suppression if their concentrations become too high. They also can have solubility problems. These solubility issues have led to the predominant use of methanol since the fluoroalcohols, in particular, are significantly more soluble relative to acetonitrile.

## Materials used in LC–MS of oligonucleotides

Oligonucleotide analysis by LC–MS can be significantly complicated by the introduction of incompatible materials into the method. Most of these issues can be categorized into two groups: nucleases and high surface-active materials. Nucleases are a class of enzymes that hydrolyze oligonucleotides and therefore will degrade samples. The greatest source of nucleases in the laboratory is the analyst. Nucleases are secreted from the skin and; therefore, it is critical to be very cognizant of the surfaces in the laboratory that have been touched. It is highly advised that specific areas of the lab be marked off for the handling of oligonucleotides. All surfaces in this area should be regularly cleaned with bleach or other reagents that can ensure that no residual nucleases remain. Personnel working in this area should be gloved at all times. Materials that are brought into the area should be autoclaved (when possible); of single use; and certified to be nuclease free. Nuclease-free water should be used when making solutions that will come into contact with oligonucleotides.

The other major issue is to avoid materials with high surface activities. These materials can become easily charged and can cause significant nonspecific adsorption losses of oligonucleotides and other acidic analytes [28–32]. Many plastics that are designed for use with oligonucleotides will generally have details about their low activity or low nonspecific binding potential. In all cases, these are highly recommended for use. The other major source will be metal-oxide surfaces found primarily in the liquid chromatography system. These can be in the injector, in-line filters, tubing and columns. The greatest single source are the column frits, which comprise over 70% of the surface area of an LC system [33,34]. Metal oxide surfaces are easily capable of becoming positively charged and have long been shown to nonspecifically adsorb acidic analytes. Oligonucleotides are particularly susceptible to this process. Recently, there have been advances involving the polymeric surface coating of these metal oxide surfaces on columns and entire LC systems. The use of such systems, have been shown to have significant positive impacts on method robustness and sensitivity [33–35].

## The impact of pH

Since oligonucleotides and most of the components of the mobile phase are ionizable, the pH will have an impact on the behavior of all facets of the separation. Oligonucleotides have two ionizable moieties, the phosphodiester backbone and the nucleobases. The phosphodiester backbone has a pKa of approximately 1 and therefore will be negatively charged at all pH’s used during the analysis of oligos. The phosphorothionate backbone, prevalent in so many therapeutic oligonucleotides has a pKa a little lower than a phosphodiester and will also be charged during all practical pH’s used during analysis of oligonucleotides.

The nucleobases are all neutral at physiological pH. The N1 position on adenosine has a pKa of 3.5 and is only charged a low pH. The N7 position of guanosine has a pKa of 1.6 and is only charged at very low pH, the N1 position of guanosine has a pKa of 9.2 and can be deprotonated at high pH values that are not uncommon in LC–MS of oligonucleotides. The N3 position of cytosine has a pKa of 4.2 and is only charged at low pH. Finally, the N3 position of uridine and thymidine have pKa’s of 9.2 and 9.7 respectively and can also be deprotonated a high pH value. Since most mobile phases for oligonucleotide LC–MS are between pH values of 8 and 10, the bases do not greatly add to the charging of the oligonucleotide. Given its acidic nature, the phosphate backbone will be the major contributor to the overall charge of an oligonucleotide. While some of the bases may contribute to charging at high pH values they are not as prevalent in the overall structure of the oligonucleotide. It is always possible that this contributes to greater nonspecific adsorption but the evidence to date suggests that nonspecific losses decrease with increasing pH and so this does not appear to be a meaningful contribution to the overall process.

The alkylamines typically used for LC–MS have pKa values between 9.5 and 10.5. Therefore, they are generally cationic which serves their main purpose in ion pairing with the phosphate backbone of the oligonucleotide. HFIP is the most widely used as a mobile phase additive and has a pKa of 8.0. It is therefore negatively charged during most LC–MS conditions, which is consistent with its role as a counterion to the alkylamine. The next most common fluoroalcohol is hexofluoromethylisoproponal (HFMIP) has similar acid/base character to HFIP with a pKa of 8.3. There are a few other fluoroalcohols that have been used but only HFIP and HFMIP appear to be ideal for LC–MS of oligonucleotides [9,36].

The final component of the LC–MS determination of oligonucleotides that needs to be mentioned are the metal oxide surfaces found throughout the LC system and column. These surfaces can be positively charged at pH ranges below 9. This causes them to adsorb oligonucleotides in a nonspecific manner. The nonspecific binding of acidic compounds to charged metal surfaces has been known for many years. Generally, this issue is overcome by passivation, which is making injections with either the analyte or more often a surrogate oligonucleotide to occupy the multitude of nonspecific binding sites in the instrument. Recent work by Gilar *et al.* has shown that it appears to take 10 s of µg of oligonucleotides to fully passivate an LC system [34]. Passivation needs to be continuously maintained because LC systems can be easily depassivated.

## High pH conditions

**Figure 1.**
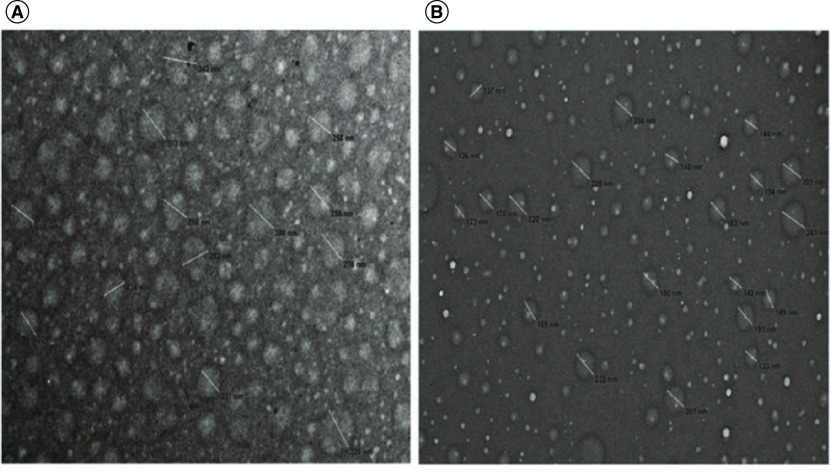
Mobile phase aggregate formation shown through transmission electron microscopy. Transmission electron microscopy images using carbon-coated nickel grids of the mobile phase containing 65 mM N,N-Diisopropylethylamine (DIEA) and 50 mM HFIP in 10% methanol heated at 60°C. **(A)** After drying at 60°C. **(B)** After drying at room temperature. Reprinted with permission from Li *et al.*, *Journal of Chromatography A* [37].

As the pH of the mobile phase rises greater amounts of the alkylamine become neutral. A study by Li and co-workers showed that under these conditions the alkylamines will begin to form micelles in the mobile phase [37]. This manifests itself in the method as a dramatic drop in analyte retention time. This loss in retention begins below the pKa of the alkylamine, so only a fraction of the alkylamine needs to be neutral to initiate this aggregation. Li and co-workers also showed that elevated column temperatures increased the formation of the micelles in the mobile phase, which was consistent with earlier work on nonionic detergents. Figure 1 illustrates aggregate formation observed through transmission electron microscopy at two different temperatures. In addition, lowering the pH to 7.5 with formic acid showed loss of aggregates in the mobile phase.

**Figure 2.**
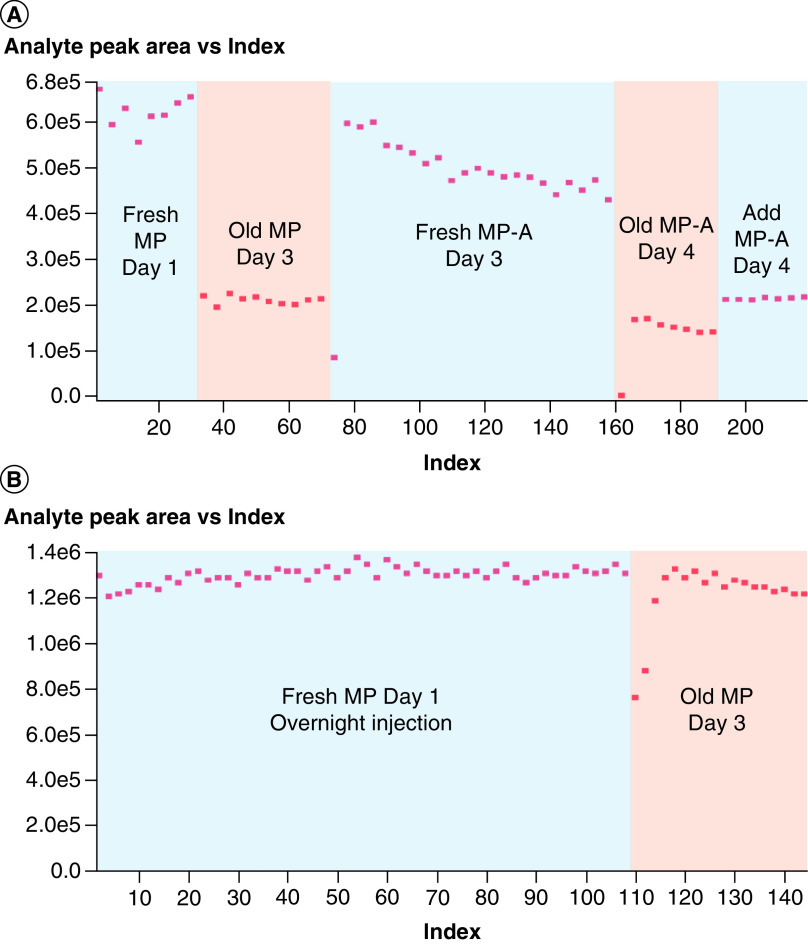
Antisense oligonucleotide mass spectrometry signal stability in different liquid chromatography system configurations. **(A)** Signal intensity plots for ASO-1 on a binary LC system, showing signal loss over days and even within the run. **(B)** Ternary LC system, showing a stable signal within a long overnight run and from a cold start after 2 days. Reprinted with permission from Li *et al.* Analytical Chemistry [39]. ASO1: Antisense oligonucleotide analyte; LC: Liquid chromatography.

This loss in method performance has been reported numerous times over the past 30 years and is referred to as mobile phase aging. It is normally expressed in papers by the frequency that mobile phases need to be remade. This has been as frequent as daily. Kim and co-workers have reported a 60% drop in signal intensity in 24 hours from a HFIP/dimethylcyclohexylamine (DMCHA) mobile phase system [38]. A recent breakthrough in increasing mobile phase stability was made by Li and co-workers who went to making a ternary mobile phase [39]. In their mobile phase the alkylamine and fluoroalcohol were placed in 100% acetonitrile rather that in a predominantly aqueous environment. The lack of water greatly reduces the formation of these micelles and reduced the frequency with which they remade their mobile phases to weekly. Mobile phase aging and performance loss can be seen in Figure 2 by tracking the response of a antisense oligonucleotide standard (ASO-1) over multiple days. The ternary pump system increases mobile phase performance substantially, suggesting that not only pH, but also the aqueous phase are contributing factors in mobile phase aging.

At higher pH values, the surface charging of metal surfaces in the chromatographic system are reduced. This decreases, but does not eliminate, the nonspecific adsorption that is present in the method [34].

## Low pH conditions

As the pH of the mobile phase decreases you approach the pKa of the fluoroalcohol. There does not appear to be any significant consequences of HFIP becoming more neutral. However, there may be subtle chromatographic alterations due to this change.

**Figure 3.**
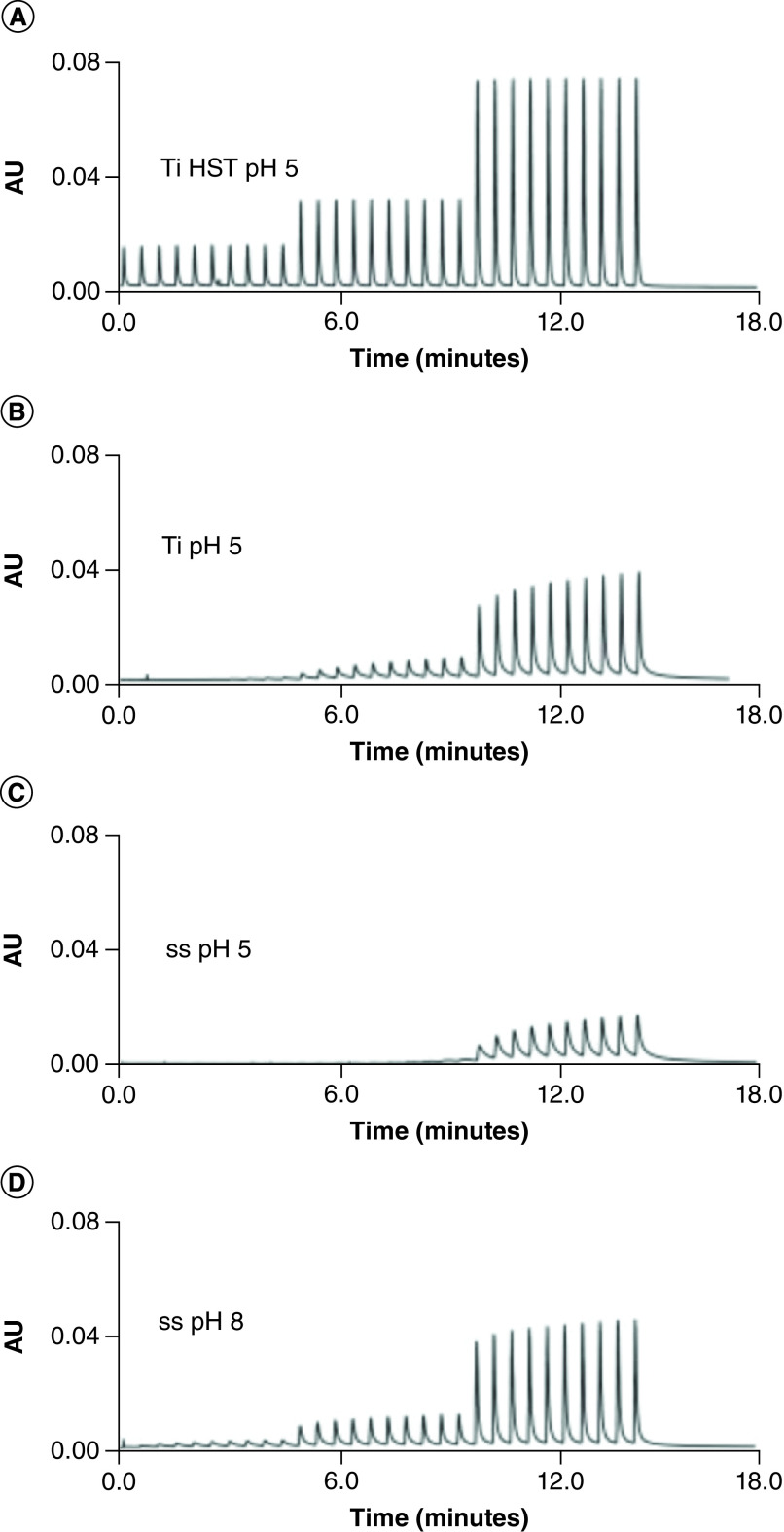
Results of multiple injection in a single run experiments consisting of 10 × 10 ng injections followed by 10 × 20 ng and 10 × 50 ng injections of adenosine 5′-(α, β-methylene) diphosphate (AMPcP) on metal frits. **(A)** 4.6 mm id titanium frit modified with HST. **(B)** Unmodified 4.6 mm id titanium frit. **(C–D)** 4.6 mm id stainless steel frit. The mobile phase was 5 mM ammonium acetate adjusted to pH 5 for experiments A–C and pH 8 for experiment D. The peaks were detected by AU at 260 nm. Reprinted with permission from Gilar *et al.*, *Journal of Chromatography A* [34]. AU: Absorbance units.

The most substantial change is the large increase in surface charging of metal surfaces within the chromatographic system. These appear to maximize around a pH of 7; although, this is an unrealistically low pH for LC–MS separations of oligonucleotides since it may require the use of strong acids, which have shown to be major ion suppressants in LC–MS of oligonucleotides [27,40]. As the surface charging increases, the capacity of these metal surfaces to nonspecifically adsorb oligonucleotides also increases. This will make it more challenging to initially passivate a system and to maintain passivation during the analysis of batches of samples [33,34]. Acidic analyte loss to metal surfaces in acidic pH conditions has been previously discussed and characterized [30,33,34,41–43]. Figure 3 compares analyte loss to different surface types, as well as to stainless steel surfaces at pH 5 and pH 8 (Figures C & D). Analyte recovery is significant decreased at lower pHs.

## Recommendations & future studies

The pH of the alkylamine/fluoroalcohol mobile phases are determined by the concentrations of the base (alkylamine) and the acid (fluoroalcohol). In most cases the alkylamine concentration is between 5 and 15 mM. This lower concentration makes it easier to adjust the pH using the fluoroalcohol and also provides optimal electrospray ionization response. Lower concentrations will lead to decreased chromatographic retention and reduced mass spectral response due to low surface concentrations of the oligonucleotides on the electrospray droplets. Higher concentrations make it harder to titrate the pH, increase the formation of micelles and cause ion suppression. The relationship ion–pair concentration and signal intensity can be seen in McGinnis and co-workers [12]. The fluoroalcohols provide optimal performance between 30 and 40 mM. However, at these levels the pH of the mobile phase will likely be greater than 9. Further increasing the concentration of the fluoroalcohol will continue to lower the pH but will increase ion suppression from this reagent.

**Figure 4.**
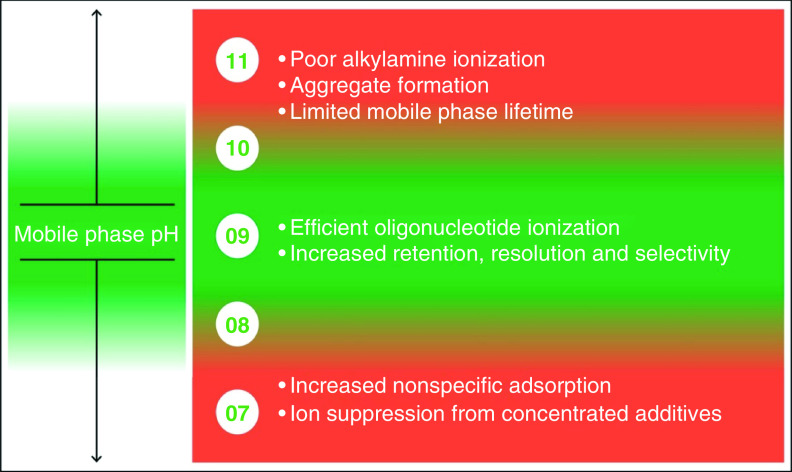
General relationship between mobile phase pH and method performance.

The need to critically evaluate the mobile phase pH is necessary in order to effectively balance method robustness and performance, and it is not straightforward. If the pH is too high, then there will be issues with chromatographic stability and mass spectral response. In addition, frequent remaking of mobile phases is time consuming and the fluoroalcohols are expensive reagents. If the pH is too low, then nonspecific adsorption will be problematic and method throughput will be impacted due to the additional time that will be needed to maintain system passivation. The final pH will be a balance between all of these factors and must take in to account the objectives of the method. A general relationship between pH and mobile phase performance can be seen in Figure 4

There still remain a number of questions that would improve our ability to more accurately define the optimal working parameters for LC–MS of oligonucleotides. The use of hybridized metal surfaces in chromatographic systems and columns is a tremendous advance in reducing nonspecific adsorption. However, it is not clear how durable these surfaces will be once deployed in R&D, nonclinical, clinical settings. The susceptibility of these surfaces to particular chemicals or biological molecules, should be closely monitored over the coming years. The rate of micelle formation is another factor that is currently unknown. Once the pH of the mobile phase reaches the pKa of the alkylamine, the formation of micelles is instantaneous. However, this formation will certainly occur below the pKa, but how quickly this will happen is not known. At the moment, it appears that keeping the mobile phase between 0.5 and 1.0 pH units below the pKa of the particular alkylamine chosen would be advisable. Also, if your chromatographic system will support ternary mobile phases, then isolating the alkylamine from water will considerably improve the stability of the method [39].
